# Dinuclear Ruthenium(II) Complexes as Two-Photon, Time-Resolved Emission Microscopy Probes for Cellular DNA**

**DOI:** 10.1002/anie.201309427

**Published:** 2014-01-23

**Authors:** Elizabeth Baggaley, Martin R Gill, Nicola H Green, David Turton, Igor V Sazanovich, Stanley W Botchway, Carl Smythe, John W Haycock, Julia A Weinstein, Jim A Thomas

**Affiliations:** Department of Chemistry, University of Sheffield Sheffield, S3 7HF (UK); Department of Biomedical Science, University of Sheffield Sheffield, S10 2TN (UK); The Kroto Institute, University of Sheffield Sheffield, S3 7HQ (UK); Central Laser Facility, Research Complex at Harwell, STFC, Rutherford Appleton Laboratory Oxfordshire OX11 0QX (UK)

**Keywords:** DNA, imaging microscopy, luminescence, ruthenium, two-photon emission imaging

## Abstract

The first transition-metal complex-based two-photon absorbing luminescence lifetime probes for cellular DNA are presented. This allows cell imaging of DNA free from endogenous fluorophores and potentially facilitates deep tissue imaging. In this initial study, ruthenium(II) luminophores are used as phosphorescent lifetime imaging microscopy (PLIM) probes for nuclear DNA in both live and fixed cells. The DNA-bound probes display characteristic emission lifetimes of more than 160 ns, while shorter-lived cytoplasmic emission is also observed. These timescales are orders of magnitude longer than conventional FLIM, leading to previously unattainable levels of sensitivity, and autofluorescence-free imaging.

Lifetime-based imaging techniques, such as fluorescent- and phosphorescent lifetime imaging microscopy (FLIM and PLIM, respectively) and time-resolved emission imaging microscopy (TREM), which combines both FLIM and PLIM, offer several advantages over conventional emission-based methods, as they facilitate measurements that are independent of probe concentration, whilst providing information on the micro-environment of the probe itself.[1, 2] Furthermore, as PLIM and TREM employ probes that emit on the hundreds of nanoseconds to microsecond timescale, these techniques negate the common problem of biomolecular autofluorescence, which typically has a lifetime in the order of a picosecond to a few nanoseconds.[3]

Although several nanosecond FLIM-based DNA probes exist[4, 5] and the number of PLIM-compatible imaging agents is increasing,[3] to date there are no reports of PLIM-compatible probes that specifically target DNA in live cells. Considering the biological significance of DNA and particularly its role in genetic disease and tumorgenesis, this is a significant current shortcoming. Furthermore, the visualization of the nucleus, one of the most distinctive organelles within a cell, is a key step within a plethora of experimental cell biology methods.

In concurrent research, methods for two-photon (2P) absorption imaging technologies are also being sought. Simultaneous absorption of two photons of low-energy light (for example, near-IR) leads to probe emission in the visible region and enables visualization of subcellular structures at submicrometer diffraction-limited resolution. Furthermore, 2P-based microscopy is particularly attractive for live cell-based samples, as low-energy excitation wavelengths within the biological optical window can be used, facilitating the possibility of luminescence-based deep tissue imaging.[6–8]

Studies into luminescent transition-metal complexes that function as biomolecular probes have attracted much attention and, more recently, this research has been extended to include studies within cells, in which the metal complex functions as an imaging agent for luminescent microscopy techniques, most commonly confocal microscopy.[9–11] Using this approach, novel probes for specific organelles and biomolecular targets have been identified.[12, 13] However, virtually all of this work exclusively involves steady-state emission where changes in intensity or emission energy of the probe are used as the imaging signal. Although recent studies have sought to understand the stability of metal complexes used as PET/SPEC imaging (PET=positron emission tomography, SPEC=single photon emission computed tomography),[14] lifetime-based imaging involving metal complexes has hardly been explored at all. This deficiency is surprising because transition-metal complexes are particularly suitable for this technique, as they emit from long-lived, triplet-based excited states that are usually efficiently populated through the heavy-atom effect. Furthermore, transition-metal complexes often possess high two-photon absorption cross-sections, making them particularly compatible with 2P-based lifetime microscopy techniques.[15] Despite this fact, there are very few reports combining multiphoton excitation with imaging on timescales longer than several nanoseconds.

Recently, multiphoton PLIM using metal complex probes has been applied to imaging of pO_2_ in mouse brain tumor in vivo. However this study used a frequency-multiplex, line-scanning approach, which does not provide the sub-micrometer spatial resolution that is essential for intracellular studies.[16] We have recently described how highly emissive, charge-neutral platinum(II) complexes previously shown to function as membrane-permeable, intracellular TREM probes for sub-cellular structures, particularly nucleoli,[17] can be imaged in live cells and histological tissues with submicrometer resolution on a time scale of up to several microseconds under two-photon excitation. This study introduced 2P-PLIM/TREM with submicrometer resolution and demonstrated that by careful control of pulsed laser photon energy, multiphoton imaging with microsecond lifetime could be compatible with live mammalian cells.[18]

Ruthenium polypyridyl complexes, which usually display intense emission in the visible region with a lifetime of hundreds of nanoseconds, have been used as steady-state sensors for a wide range of targets, particularly biomolecules. For example, apart from their use in imaging duplex DNA and RNA, probes for a variety of non-canonical nucleic acid structures have been developed.[19, 20] Probes in cells for non-nucleic acid structures, including the membranes that define the endoplasmic reticulum,[21] as well as peptide and protein aggregates associated with neurodegenerative diseases,[22, 23] have also been reported. Furthermore, as the previously mentioned pO_2_ study and related work demonstrates, ruthenium polypyridyl complexes offer great potential as lifetime-based probes.[16, 24] This is further illustrated by a 1P-PLIM study in which Ru(dppz) systems were used to probe the lipophilicity of cellular microenvironments.[25]

We have previously reported on the cellular uptake properties of the two dinuclear Ru^II^(tpphz) compounds **1** and **2** (Scheme 1).[26] Through a “DNA light-switch” effect,[27] they are essentially non-emissive in aqueous environments but display bright luminescence when bound to DNA, thus minimizing any image contrast problems that are due to emission from non-bound luminophores. The chloride salts of dinuclear cationic complexes **1** and **2** display excellent water solubility and bind to DNA with high affinities (>10^7^ L mol^−1^) through a non-intercalative mechanism.[21, 28] Furthermore, confocal microscopy studies involving both complexes have revealed that complex **1** in particular is actively transported into the nuclei of live cells, where it then stains chromatin.[26] As the luminescence of **1** and **2** is from a long-lived Ru→L ^3^MLCT (metal-to-ligand charge-transfer) state,[29] the light-switch effect leads to increases in both emission intensity and emission lifetime. Herein we demonstrate that these properties mean that **1** and **2** also function as DNA specific lifetime-based intracellular probes, where visualization is achieved through long emission lifetime on time scales more than 10 times longer than the fluorescence of usual organic labels, with two-photon (2P) excitation providing sub-micrometer spatial resolution. Using this approach, 2P-PLIM based imaging of DNA at lifetimes of more than 160 ns is made possible, totally removing any possibility of crosstalk from endogenous fluorophores. This is the first time that phosphorescence lifetime imaging, which also offers autofluorescence-free visualization, has been applied to image cellular DNA and whole chromosome structures.

**Scheme 1:**
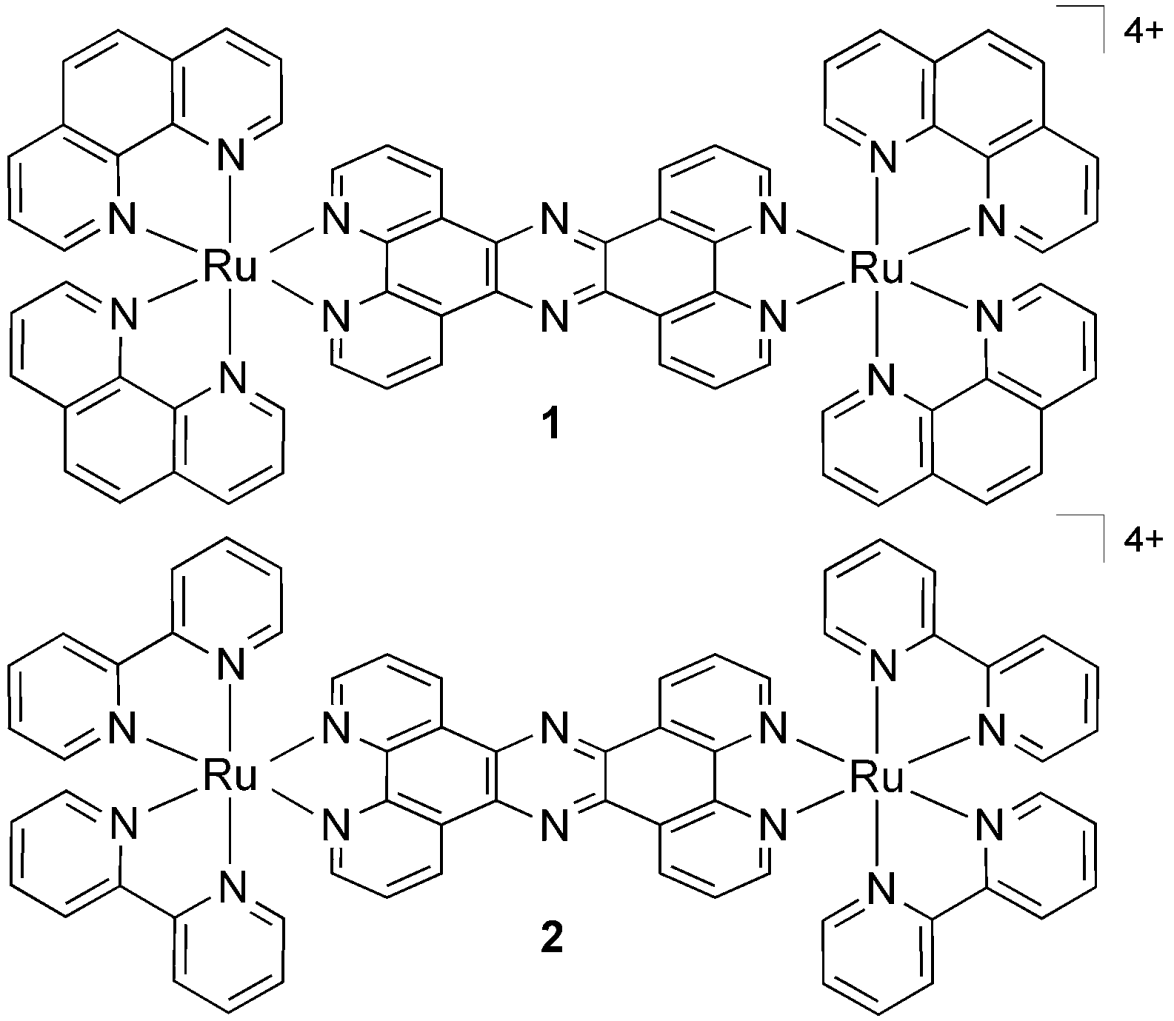
Structures of the complexes used in this study.

First, the suitability of **1** and **2** for PLIM under two-photon excitation is confirmed by their appreciable two-photon absorption cross-sections, which are found to be 142 GM (Goeppert–Mayer) and 108 GM (±24 %), respectively (see the Supporting Information). As we have previously demonstrated that **1** is rapidly internalized by MCF-7 human breast cancer cells, where it targets nuclear DNA,[26] identical experimental conditions were employed as a starting point for the following imaging studies.

2P-PLIM images and representative decay kinetics from live MCF-7 cells labeled with **1** are presented in Figure 1. Predominantly emission is observed from the cell nuclei, which exhibits a lifetime of 165±16 ns (left). Focusing upon a single cell (right) from the original field of view provides a similar emission lifetime from the nucleus (176±11 ns). Not only are these lifetimes in excellent agreement with one another, they are also in good agreement with lifetime data from the nucleii of HaCat human keratinocytes cells similarly labeled with **1** (*τ*: 175±6 ns; Supporting Information, Figure S1).

**Figure 1:**
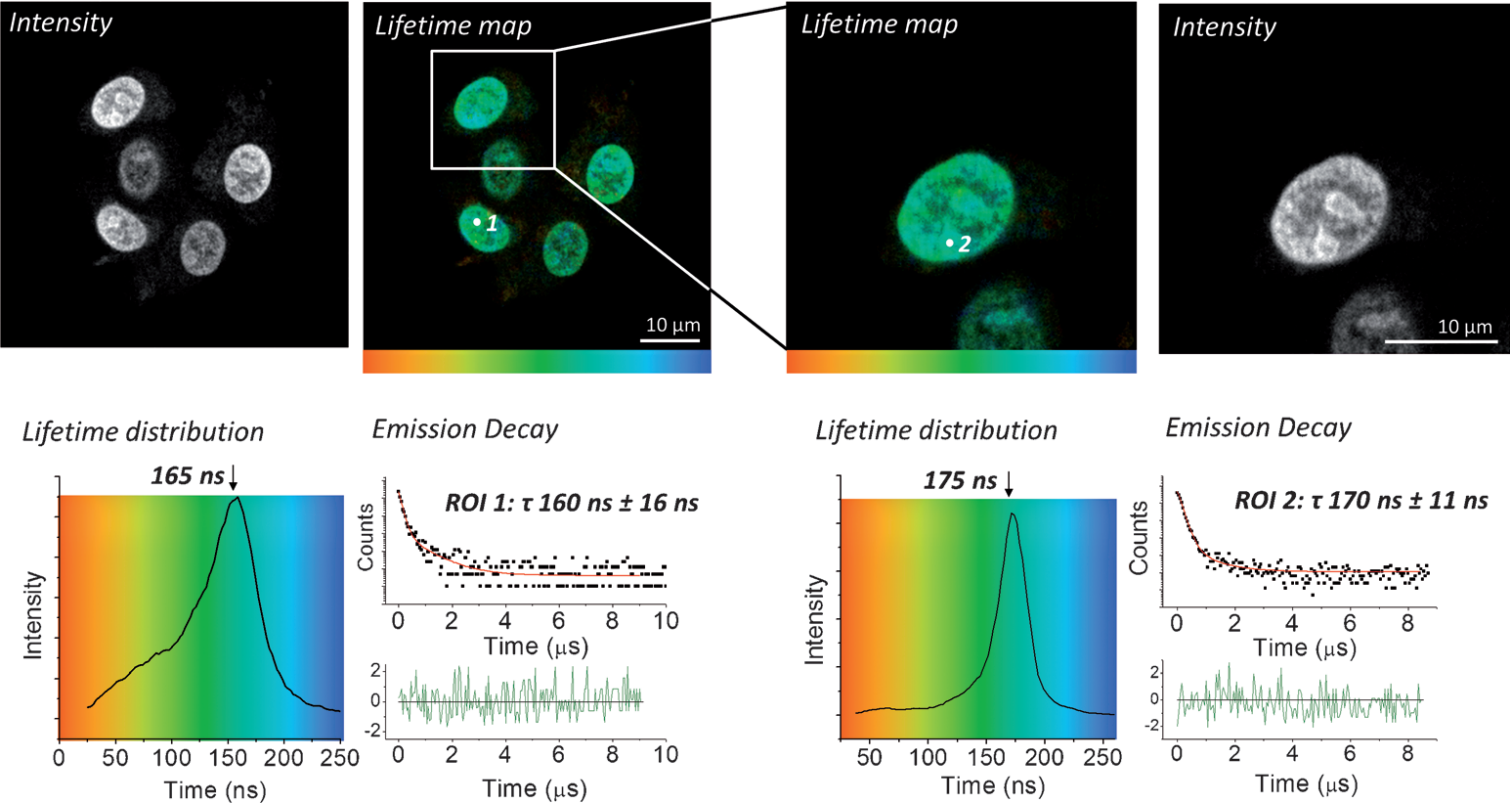
PLIM imaging of live MCF-7 cells pretreated with complex **1** (500 μm, 1 h, serum-free media).

To further investigate the capability of **1** and **2** to act as high-resolution 2P-PLIM DNA stains, metaphase spreads of HeLa human cervical cancer cell chromosome were prepared. This technique involves trapping of cells in the metaphase stage of mitosis and spreading chromatin onto slides to allow the clear visualization of the condensed chromosomal DNA. As shown by Figure 2 A, addition of **1** or **2** to the chromosomal spreads produces high-contrast confocal images of cellular DNA in sister chromatids, which are imaged in detail. Again, similarly striking PLIM lifetime and intensity images of the chromosomes are observed (Figure 2 B). Lifetimes recorded from both the metaphase and interphase nuclei (182±12 ns and 182±4 ns, respectively) are again in excellent agreement with one another and with those recorded from live MCF-7 cells. This indicates that **1** is in the same micro-environment in all cases and bound to DNA in the same way. The small region of short-lived emission (Figure 2 B, location c) is due to complex precipitate. While this is not endogenous to the biological system, it serves as an example of the ability of the technique to distinguish between regions of different emission lifetimes in the same field of view. Similar results were observed for complex **2** under the same experimental conditions, where lifetimes of 175±4 ns and 168±7 ns for interphase and metaphase nuclei respectively were recorded (Supporting Information, Figure S3).

**Figure 2:**
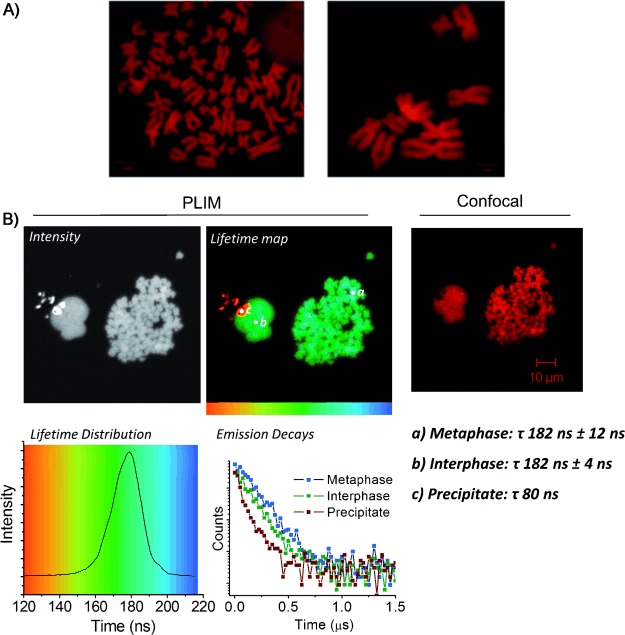
A) Metaphase spreads of HeLa chromosomes stained with **2** (left) or **1** (right) (100 μm, 30 min) and imaged by confocal microscopy; B) 2P-PLIM (left) and confocal (right) imaging of HeLa metaphase spreads labeled with **1**.

We have previously established that the mechanism by which **1** achieves nuclear targeting in live cells is by active transport and that **2** displays poorer live cell uptake.[26] Therefore, to further characterize the intracellular distribution of these probes in the absence of specific uptake mechanisms, PLIM imaging of formaldehyde-fixed and Triton-permeabilized MCF-7 cells stained with **1** or **2** was carried out.

The comparison between 2P-PLIM and images acquired by standard confocal microscopy for **1** are shown in Figure 3. Emission from complex **1** within cell nuclei is clearly observed using both imaging methods. A nuclear lifetime of 185±12 ns (position a, blue region) is again consistent with a DNA bound complex, as observed in live cells and fixed metaphase spreads. Importantly, while cytoplasmic staining appears significantly less intense than nucleic staining by confocal microscopy, 2P-PLIM allows this phenomenon to be explored in more detail. A shorter lived cytoplasmic emission of 124±13 ns (position b, yellow–green region) for complex **1** is also observed, clearly indicating that this complex is present outside the nucleus. These data are consistent with HaCat keratinocyte cells similarly labeled with **1**, where lifetimes of 195±6 ns and 109±10 ns are observed for **1** in nuclear and cytoplasmic staining respectively (Supporting Information, Figure S4). In formaldehyde-fixed cells under these conditions, **2** displays similar properties to **1** in both cell lines, with nuclear lifetime of 190±10 ns, and a shorter cytoplasmic lifetime of 150±10 ns (Supporting Information, Figure S5 and S6). While not initially apparent in live cells owing to the high-intensity nuclear emission, a shoulder (ca. 100 ns) visible in the lifetime distribution histogram (Figure 1, left) indicates the presence of a shorter-lived component. Closer inspection reveals this signal is due to a minor amount of cytoplasmic staining, and increased pixel binning facilitates fitting of the decay data, revealing a cytosolic lifetime value of 110±13 ns (Supporting Information, Figure S2).

**Figure 3:**
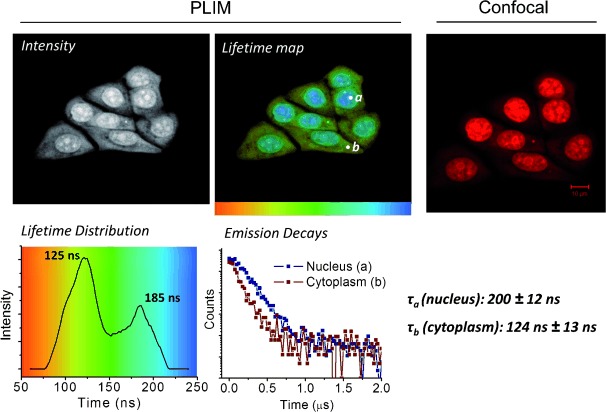
PLIM (left) and confocal (right) comparison of fixed, permeabilized MCF7 cells treated with complex **1** (100 μm, 45 min, PBS buffer).

These emission lifetime measurements imply that the micro-environment of the cytoplasmic bound complex, where it is most likely bound to cytosolic proteins, is different to that of the nucleus. The longer nuclear lifetime indicates that protection from surrounding water molecules is more effective when **1** is bound to DNA. This also explains why cytosolic emission is difficult to observe though confocal microscopy, as increased quenching results in a reduction in quantum yields and emission intensities. These observations suggest that the range of intracellular targets of complexes **1** and **2** is broader than can be determined by simple fluorescence microscopy alone and are consistent with previous reports on structurally related mononuclear dppz-derived complexes, which also exhibit longer emission lifetimes when DNA-bound.[25]

In conclusion, in this initial study using ruthenium(II) luminophores, we present the first 2P-PLIM imaging probes for nuclear DNA in both live and fixed cells. The DNA-bound probes display a characteristic emission lifetimes of more than 160 ns, while a shorter-lived cytoplasmic emission is also observed. These timescales are orders of magnitude longer than conventional FLIM and thus provide high-sensitivity autofluorescence-free imaging.

Given the distinctively different lifetimes observed for nuclear and cytosolic location, future studies will focus on the possibility that these complexes interact with other biological targets within the cytosol, such as specific proteins and RNA structures, which cannot be imaged by conventional luminescence methods. Within the nucleus, the ability of these probes to report lifetime differences will facilitate novel investigations of chromatin micro-environment under a variety of physiological circumstances, including proliferation, quiescence, senescence, and cell cycle progression.

As both these complexes display good water solubility, low toxicity, and **1** is particularly well taken up by active transport into living cells, their application in 2P-PLIM provides a new modality for DNA targeting that can be potentially extended to provide an array of probes for specific sub-cellular targets, even in deep tissue.

## Experimental Section

**1** and **2** were synthesized and characterized as described previously[29a] and used as their chloride salts. Each compound was used as a mixture of enantiomers. 2P-PLIM imaging was performed using Becker & Hickl GmbH combined FLIM/PLIM apparatus connected to a Zeiss-510 Meta Microscope. PLIM data was processed using SPCImage software. See the Supporting Information for full details of experimental apparatus, conditions, and procedures.

## Supporting Information

As a service to our authors and readers, this journal provides supporting information supplied by the authors. Such materials are peer reviewed and may be re-organized for online delivery, but are not copy-edited or typeset. Technical support issues arising from supporting information (other than missing files) should be addressed to the authors.

miscellaneous_information
