# Supplementary material for: Dinuclear Ruthenium(II) Complexes as Two-Photon, Time-Resolved Emission Microscopy Probes for Cellular DNA
Source: Angew Chem Int Ed Engl. 2014 Jan 23;53(13):3367–71. doi: 10.1002/anie.201309427 (PMC4298790; doi:10.1002/anie.201309427)
Supplement: Supplementary file 1 — miscellaneous_information [file anie0053-3367-SD1.pdf]

Angewandte Chemie
Eine Zeitschrift der Gesellschaft Deutscher Chemiker

Supporting Information

© Wiley-VCH 2014

69451 Weinheim, Germany

Dinuclear Ruthenium(II) Complexes as Two-Photon, Time-Resolved Emission Microscopy Probes for Cellular DNA**

Elizabeth Baggaley, Martin R. Gill, Nicola H. Green, David Turton, Igor V. Sazanovich, Stanley W. Botchway, Carl Smythe, John W. Haycock, Julia A. Weinstein, and Jim A. Thomas**

anie_201309427_sm_miscellaneous_information.pdf

Preparation of complexes:
1 and **2** were synthesized and characterized as described previously [1] and converted to their chloride salts by anion metathesis.

Cell culture:
MCF7 cells were cultured in a humidified 37°C, 5% CO_2/95% air (v/v) environment in RPMI1640 (Roswell Park Memorial Institute) media supplemented with 10% FBS (fetal bovine serum) and penicillin/streptomycin.
HaCat cells were cultured in a humidified 37°C, 5% CO_2/95% air (v/v) environment in Dulbecco's Modified Eagle's Medium (DMEM, Sigma-Aldrich) supplemented with 10% (v/v) FCS (fetal calf serum), L-glutamine (200 mM, 5 mL), penicillin/streptomycin (5mL), fungizone (1.25 ml).
Both cell lines were cultured as monolayers in T-75 flasks and passaged using trypsin-EDTA. For imaging experiments cells were seeded in to 6 well plates and cultured until 60% confluent.

Staining with Ruthenium complexes
Live cells: After removal of growth media cells were washed with PBS (phosphate buffered saline) (1 ml/well) before treating with a solution of **1**; *i)* 500 µM in serum free RPMI (1h at 37°C, 1ml/well) for MCF7 cells and *ii)* 500 µM in full DMEM (2h at 37°C, 1 ml/well) for HaCat cells. After incubation with **1**, cells were washed with more PBS (3 x 1 ml/well) to remove excess complex before imaging.

Fixed cells*:* After removal of growth media cells were washed with PBS (1 ml / well) before fixing with 10 % formaldehyde in PBS (30 min at rt, 1 ml/well). Cells were then permeabilised using 0.1 % Triton X in PBS (10 min at rt, 1 ml/well) before staining with **1** or **2**; *i)* 100 µM in PBS (45 min at rt, 1 ml/well) for MCF7 cells *ii)* 500 µM in PBS (1h at rt, 1 ml/well) for HaCat cells. Cells were washed with PBS (3x 1 ml/well) to remove excess ruthenium(II) complex before imaging.

Metaphase spreads: Hela cells were treated with 0.05µg/ml Colcemid (Sigma) for 4 hrs at 37°C, Trypsinsed and incubated in 0.056M KCl for 20 min at 37°C. Cells were fixed in methanol/acetic acid (3:1) and spread onto glass slides, air dried overnight and fixed in 4% formaldehyde/PBS. Slides were treated with 100µg/ml RNase A for 1h at 37°C prior to serial dehydration in 70%/85%/100% ethanol. Slides were stained with **1** or **2** (100 µM, 30 min) and washed with PBS.

Confocal and Time-resolved imaging:
Live and fixed cells were imaged in PBS (in 6 well plates) using a x40 water dipping objective. Metaphase spreads were imaged on mounted glass slides using either a x40 or x63 oil immersion objective.

Time-resolved, phosphorescence lifetime imaging (PLIM) of cells and metaphase spreads was carried out using a Ti:Sa pulsed laser (λ_{ex}: 800 nm) on a LSM 510 upright confocal microscope connected to a Becker and Hickl combined FLIM/PLIM system, that is comprised of a SPC-150 TPSPC module and a DDG-210 pulse generator module.[2] PLIM images were recorded using the 12 µs predefined set-up on the SPCM software (laser on time: 2 µs, PLIM decay window: 12 µs) and data processed using SPCImage software, with a pixel bin of 3 unless stated otherwise. Decay traces were best fit to a double exponential decay model (in all cases), thus emission lifetimes are reported as the τ_m average. Confocal images were recorded using standard setting on the LSM 510, λ_{ex}: 458 nm / λ_{em}: 670-700 nm.

Determination of two-photon absorption cross-section:
The two-photon absorption cross-section for **1** and **2** were measured by comparative technique, using Rhodamine B in MeOH as a reference [3] on a bespoke, two-photon laser scanning microscope, constructed in the Central Laser Facility of the Rutherford Appleton laboratory. Emission spectra under two-photon excitation were recorded by sending the emission signal from a specific pixel position through a port on the microscope to the detection setup composed of Acton 275 spectrograph and a CCD (Andor iDUS).

A MeCN solution of ruthenium(II) complex (complex **1**: 1.95×10^{-4} M, complex **2**: 1.82×10^{-4} M) in a glass-bottom dish was placed into the sample holder of the laser scanning microscope. The beam was parked in the centre of the field of view and focused near the bottom of the dish. The excitation power was varied in the broad range up to the saturation limit of the sample, and the number of emission spectra from the sample solution was taken at different power settings. A set of emission spectra from the reference solution of Rhodamine B in MeOH (3.32×10^{-4} M) was taken immediately after that under identical conditions.

The emission intensity (F) for sample and reference was integrated in the range: *i)* 608-613 nm for complex **1** and *ii)* 616-621 nm for complex **2**, where the normalised emission curves of the sample and reference overlapped. F was plotted against squared power (W^2) of the excitation laser beam to determine the power range where the dependence is linear (i.e. before the sample signal saturates). The slope of this dependence (b) was obtained by a linear fit of F vs. W^2.

The value of two-photon absorption cross-section was calculated according to modified equation presented by Rebane and co-workers:[3]

$\sigma_s = \sigma_r b_s c_r \varphi_r/(b_r c_s \varphi_s)$ (1)

where σ is the two-photon absorption cross-section, b is the slope of linear dependence of F vs. W^2, c is the molar concentration, and φ is the differential emission quantum yield in the spectral range: *i)* 608-613 for **1** and *ii)* 616-621 nm for **2**. The differential emission quantum yield of Rhodamine B, was calculated using both intervals for the two separate calculation. The subscript *s* or *r* means either sample or reference. The value σ_r for Rhodamine B in MeOH was taken as 95 GM at 760 nm.[3] The differential quantum yield φ was obtained on a Jobin-Yvon Fluoromax 4 fluorimeter under one-photon excitation.

Firstly, the total emission quantum yield was measured for aerated solutions of both ruthenium complexes in MeCN, using $Ru(bpy)_3Cl_2.6H_2O$ in water as a reference (QY = 0.04[4]). The differential emission quantum yield φ in the spectral range: *i)* 608-613 nm for **1** and *ii)* 616-621 nm for **2,** was calculated by multiplying the total quantum yield by the fraction of emission intensity in the integrated ranges with the respect to the intensity of entire emission spectrum.

The same was done for Rhodamine B in MeOH for both regions of interest (608-613 nm and 616-621 nm). However, in order to achieve good spectral agreement for the reference under one- and two-photon excitation, a lower solution concentration required for single photon measurements.

The emission maxima and QY of Rhodamine B in MeOH is known to vary with sample concentration.[5] Therefore, the two-photon absorption cross section of the two ruthenium dimers were calculated using Rhodamine B QY which corresponds to the solution used *i)* for two-photon measurements (QY = 0.4[5]) and *ii)* for single photon measurements (QY= 0.65[5]). An average of *i)* and *ii)* is reported for each ruthenium dimer, errors corresponding to the difference between the two calculations are approximately 24%.

[1] J. Bolger, A. Gourdon, E. Ishow and J.-P. Launay, *Inorg. Chem.*, 1996, **35**, 2937.
[2] Becker & Hickl GmbH, Combined Fluorescence and Phosphorescence Lifetime Imaging (FLIM / PLIM) with the Zeiss LSM 710 NLO Microscopes, Application note. Available on www.becker-hickl.com.
[3] N. S. Makarov, M. Drobizhev, A. Rebane, *Optics Express,* 2008, **16**, 4029.
[4] K. Suzuki, A. Kobayashi, S. Kaneko, K. Takehira, T. Yoshihara, H. Ishida, Y. Shiina, S. Oishic, S. Tobita *Phys. Chem. Chem. Phys*., 2009, **11**, 9850.
[5] C. V. Bindhu, S. S. Harilal, G. K. Varier, R. C. Issac, V. P. N. Nampoori, C. P. G. Vallabhan., *J. Phys. D: Appl. Phys*., 1996, **29,** 1074.

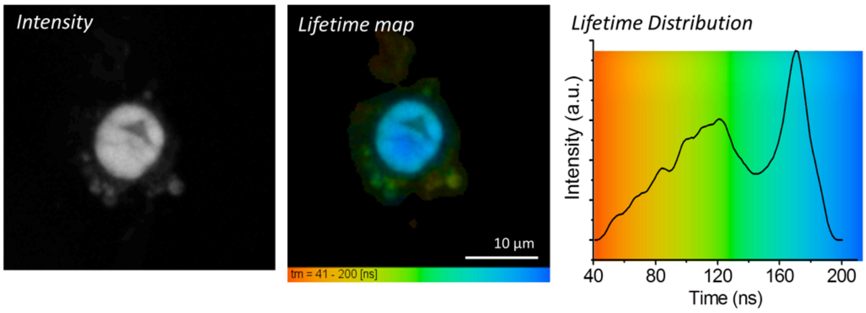

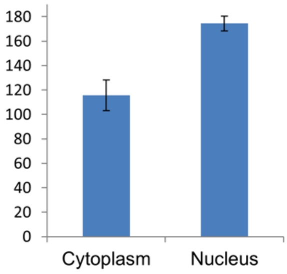

Mean lifetimes of nuclear and cytoplasmic emission of live HaCat cells stained with complex 1. Data fitted with an increased pixel bin (5) used to improve fit for low photon counts in cell cytoplasm.

Lifetimes (τ_m) taken from 10 pixels at random for each data set). Error bars represent standard deviation of each 10 point data set.

Supporting Figure S1: PLIM imaging and lifetime distribution histogram of live HaCat cells stained with complex 1 (500µM, 2hr, serum free RPMI media).

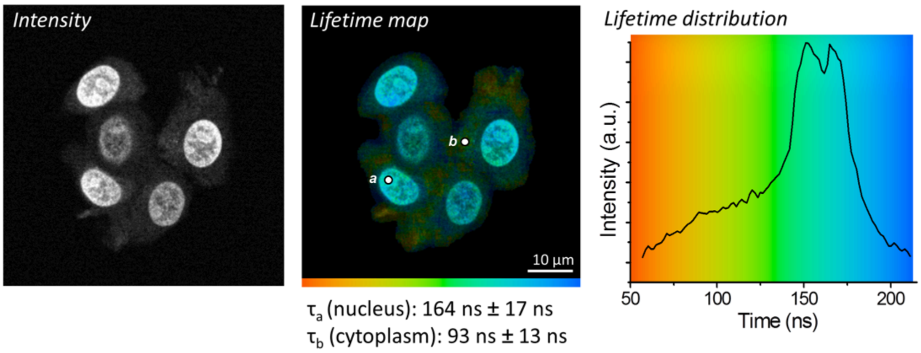

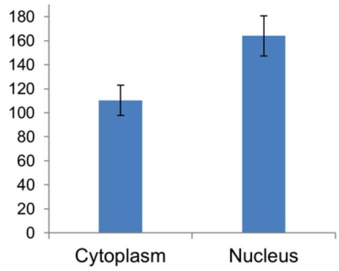

Mean lifetimes of nuclear and cytoplasmic emission of live MCF7 cells stained with complex 1. Data fitted with an increased pixel bin (7) used to improve fit for low photon counts in cell cytoplasm.

Lifetimes (τ_m) taken from 10 pixels at random for each data set). Error bars represent standard deviation of each 10 point data set.

Supporting Figure S2: PLIM imaging of live MCF7 cells pretreated with complex **1** (500µM, 1h, serum free media). Data fitted with high bin (7) to show cytoplasmic Ru emission.

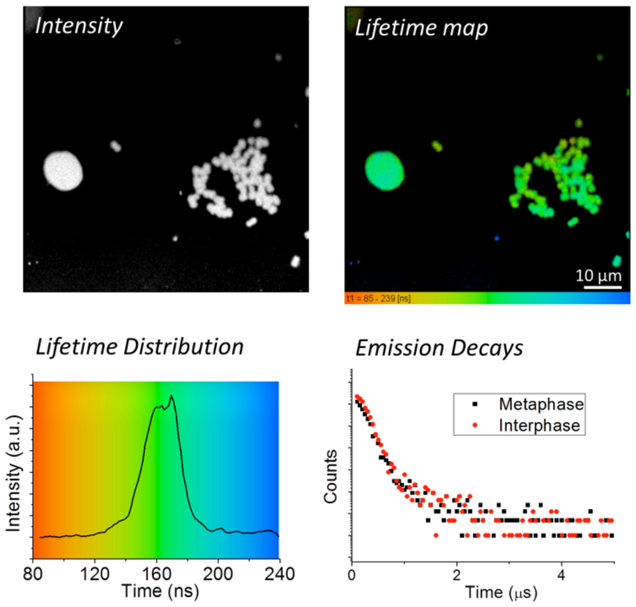

Supporting Figure S3: PLIM imaging and lifetime distribution of HeLa metaphase spreads stained with complex 2 (100 µM, PBS).

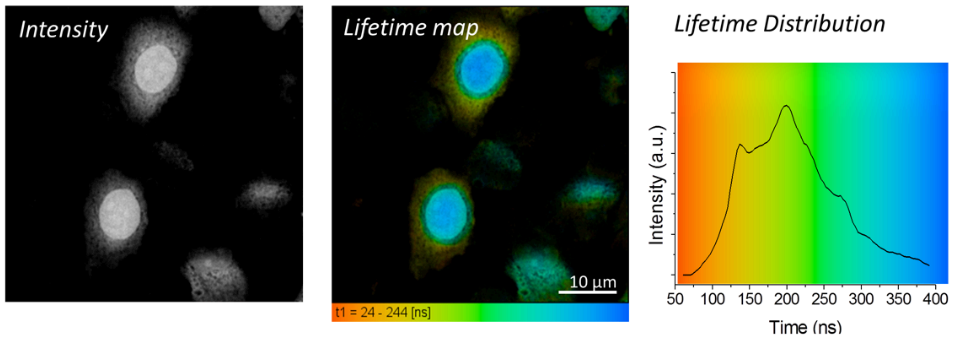

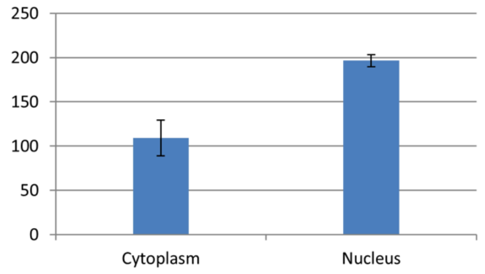

Mean lifetimes of nuclear and cytoplasmic emission of fixed HaCat cells stained with complex 1 (lifetimes taken from 10 pixels at random for each data set). Error bars represent standard deviation of each 10 point data set.

Supporting Figure S4: PLIM imaging and lifetime distribution histogram of Fixed HaCat cells stained with complex 1 (100µM, PBS).

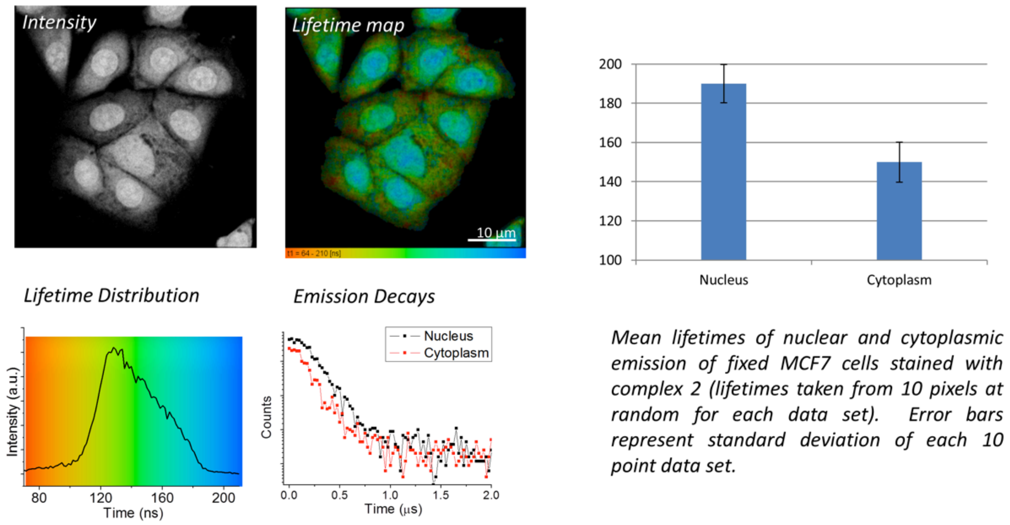

Supporting Figure S5: PLIM imaging and lifetime distribution of Fixed MCF7 cells stained with complex 2 (100 μM, PBS).

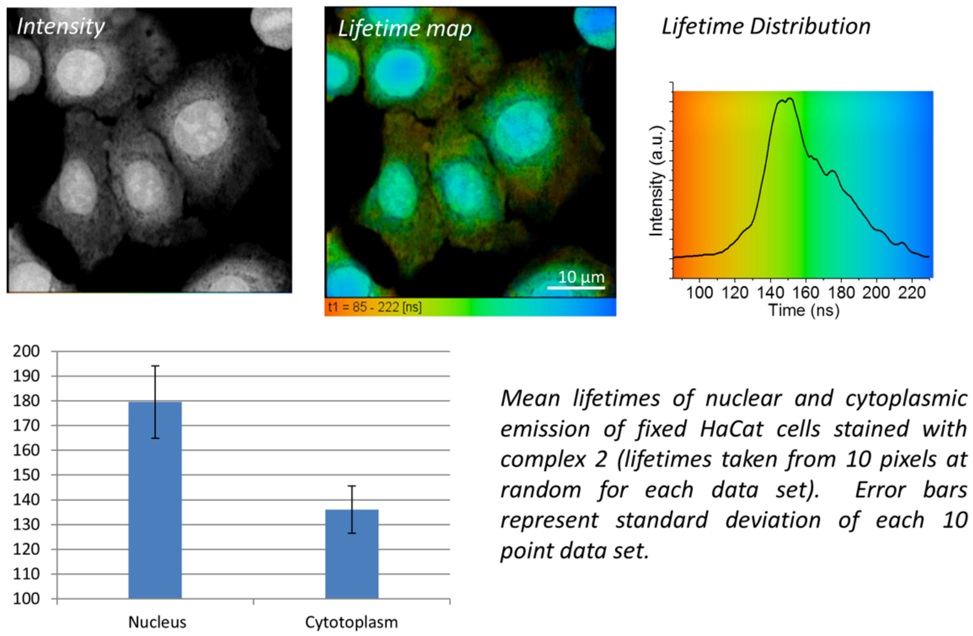

Supporting Figure S6: PLIM imaging and lifetime distribution histogram of Fixed HaCat cells stained with complex 2 (100μM, PBS).
